# Personal

**Published:** 1875-05

**Authors:** 


*Personal.*

We are under many and continued obligations to our valuable co-laborer, Dr. H. B. Lee, of Atlanta, in asisting us to make the RECORD, in every respect, all that our patrons and the public generally desire it to be. Evidences of his skillful and appreciated work will be found in this issue, and we trust that his able pen will continue its labor for the interest and edification of the reading public.

Dr. Lee's example we hope will inspire other gifted friends of the RECORD, to aid us in like manner. The object of the RECORD is to do good, and enhance the best interests of the profession, and the well being of society. In that spirit we request the use of the best talent in the medical profession, and shall always be glad to bring its thought before the world in as condensed form as possible.

